# Darier Disease – A Clinical Illustration of Its High Variable Expressivity

**DOI:** 10.7759/cureus.6292

**Published:** 2019-12-04

**Authors:** Cristina Beiu, Calin Giurcaneanu, Mara Mihai, Liliana Popa, Robert Hage

**Affiliations:** 1 Oncologic Dermatology, Elias Emergency University Hospital, Carol Davila University of Medicine and Pharmacy, Bucharest, ROU; 2 Otolaryngology, St. George's University School of Medicine, St. George's, GRD

**Keywords:** darier disease, dyskeratosis follicularis, darier–white disease

## Abstract

Darier disease (DD), also known as dyskeratosis follicularis, is a rare genodermatosis classically characterized by persistent hyperkeratotic papules and plaques affecting the seborrheic areas. Due to its variable expressivity, it can present with very discrete clinical features for many years, leading to diagnostic errors and incorrect treatments.

We report an unusual case of Darier disease in a 69-year-old male patient in which the classical features of DD had a remarkably late onset. This patient had a several decades’ history of small, recurrent, scattered papules, limited to the face, for which he had received multiple diagnostic interpretations, such as acne or recurrent staphylococcal skin infection. We established the diagnosis of DD with superinfected lesions, and initiated treatment with intravenous antibiotics and oral retinoids. Results were extremely satisfactory in a very short time.

This case shows an extremely unusual clinical course of Darier disease and is illustrative for the variable expressivity of the disease. It highlights the need to take dyskeratosis follicularis into account in patients with a longstanding history of persistent, hyperkeratotic papules, from unknown origin, even in the absence of the classical clinical findings.

## Introduction

Darier disease (DD), or Darier-White disease, is an autosomal dominant inherited skin disorder, with complete penetrance and variable expressivity. It is caused by mutations in the ATP2A2 gene, encoding the sarcoplasmic/endoplasmic reticulum Ca2+ ATPase isoform 2 protein (SERCA2) [1]. In keratinocytes, SERCA2 acts as a calcium ion pump with an essential role in the normal intracellular signaling of calcium. Keratinocytes are held together through calcium-dependent desmosomes, so the alteration of SERCA2 function causes abnormalities in keratinocytes adhesion (acantholysis) and differentiation (dyskeratosis) [2].

The disease is classically characterized by extensive persistent eruption of hyperkeratotic papules and plaques affecting the seborrheic areas, nail abnormalities and mucosal involvement. The majority of patients typically develop the initial lesions during the second decade of life [3]. Men and women are equally affected, with a prevalence of one to four per 100,000 [4].

We report an unusual case of Darier disease in a 69-year-old male patient in which the classical features of DD had a remarkably late onset.

## Case presentation

**Figure 1:**
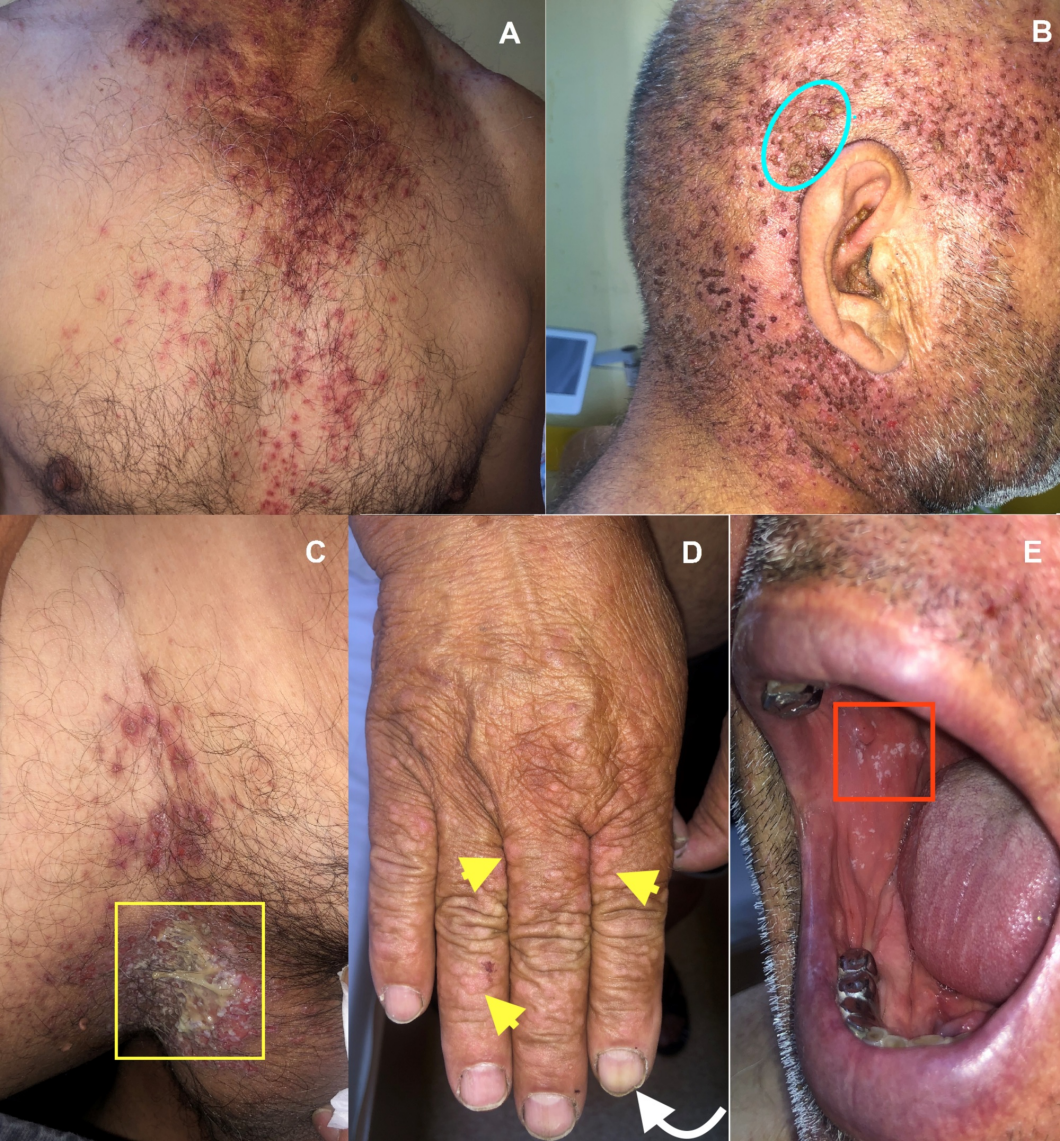
Clinical aspects of the lesions. (A) Multiple red-brown, hyperkeratotic papules, with crusted centers, involving the chest. (B) Some lesions confluated into larger, crusted plaques on the scalp (blue outline). (C) Involvement of the genito-crural folds, with maceration and large vegetative plaques in the groin (yellow square). (D) Skin-colored, warty, flat-topped papules on the dorsa of the hands (yellow arrowheads); Subtle nail changes with V-shaped notch at the distal nail edge of the index finger (white arrow). (E) Oral mucosa lesions consisted of cobblestone white papules with a central depression (red square).

A 69-year-old man presented to our dermatology department with a widespread rash consisting of multiple brown to erythematous, keratotic papules, with crusted centers, involving seborrheic areas such as scalp, ears, lateral aspects of the neck and chest (presternal area). Some lesions were scattered individually and others were confluated into larger, crusted plaques (Figure 1A, 1B). The patient also presented flexural involvement, with maceration and large vegetative plaques in the genito-crural folds, associated with malodour (Figure 1C). The lesions were itchy and the patient complained about the disfigurement, pain and malodour of the intertriginous lesions. Physical examination also showed acral abnormalities and oral involvement. Skin-colored, wart-like, asymptomatic, flat-topped papules were noticed on the dorsa of the hands (Figure 1D). The nail changes were subtle with a few discrete white and red alternating longitudinal bands and notching of the distal nail plates (Figure 1D). Oral lesions consisting of white cobblestone papules with a central depression were observed on the buccal mucosa, bilaterally (Figure 1E).

The widespread rash developed two years ago, with no obvious identifiable trigger. The patient reported that starting his mid-twenties he noted the occurrence of small scattered papules limited to the face. Along the years he presented to several dermatology clinics and received treatment for acne or recurrent staphylococcal skin infection. The lesions improved with various topical treatments, but always recurred and in the ensuing two years, the eruption continued to progress in the seborrheic and flexural areas, worsening dramatically.

The clinical aspect was suggestive of Darier disease. The patient couldn’t provide a family medical history and no other member of his family was available for analysis.

**Figure 2:**
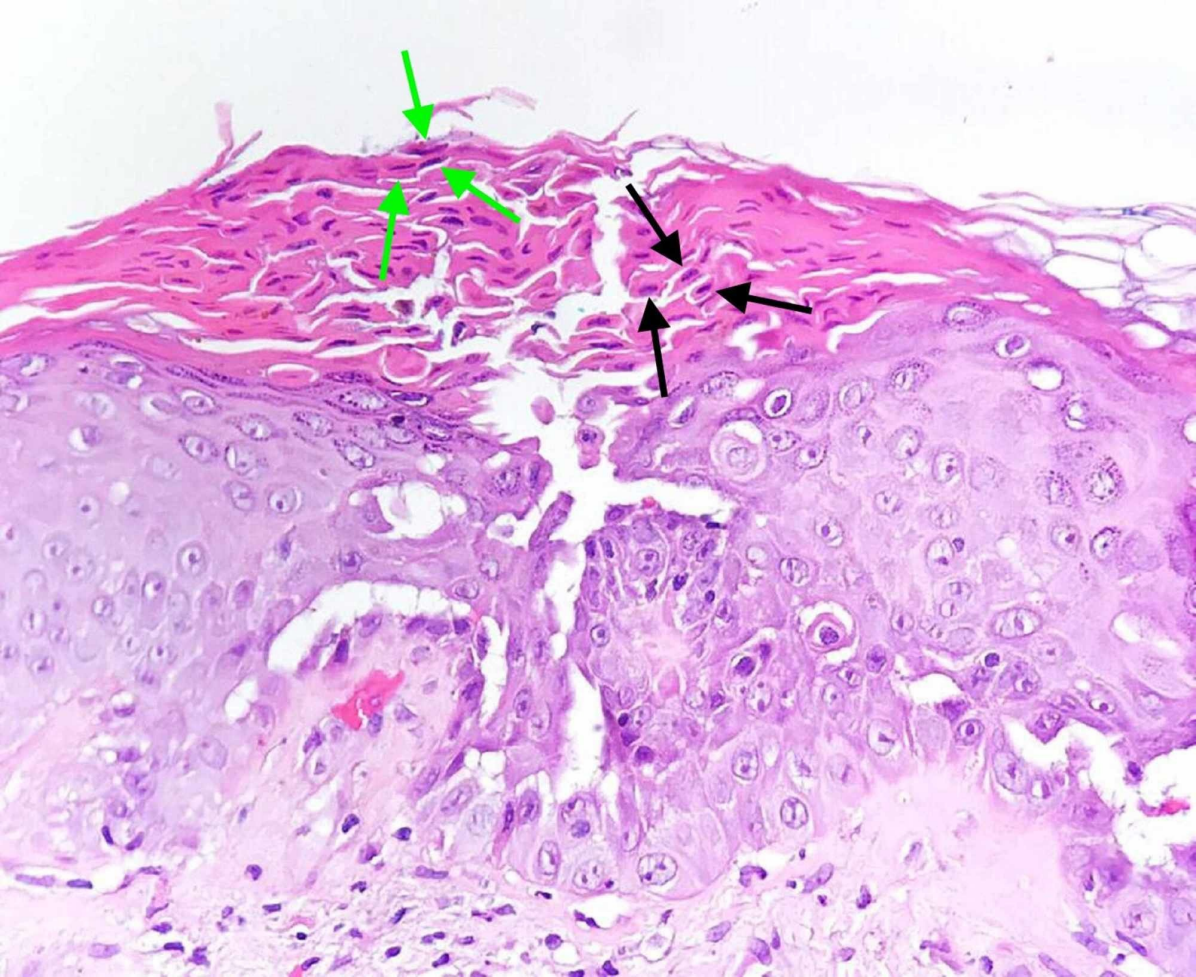
Histopathological aspect of a cutaneous fragment with focal suprabasal acantholysis and dyskeratosis with characteristic “corps ronds” - round, large, acantholytic keratinocytes predominantly located in the stratum spinosum and stratum granulosum (black arrows) and “grains” - elongated, small, acantholytic cells, mostly located in the stratum corneum (green arrows).

Routine laboratory tests were within normal limits. A punch biopsy of a hyperpigmented keratotic papule on the patient’s trunk showed focal acantholytic dyskeratosis - “corps ronds” (round, large, acantholytic keratinocytes predominantly located in the stratum spinosum and stratum granulosum) and “grains” (elongated, small, acantholytic cells, mostly located in the stratum corneum) (Figure 2). Bacterial and fungal cultures of samples from the genito-crural folds were positive for yeast (Candida albicans), as well as for Pseudomonas aeruginosa and methicillin-sensitive Staphylococcus aureus.

**Figure 3:**
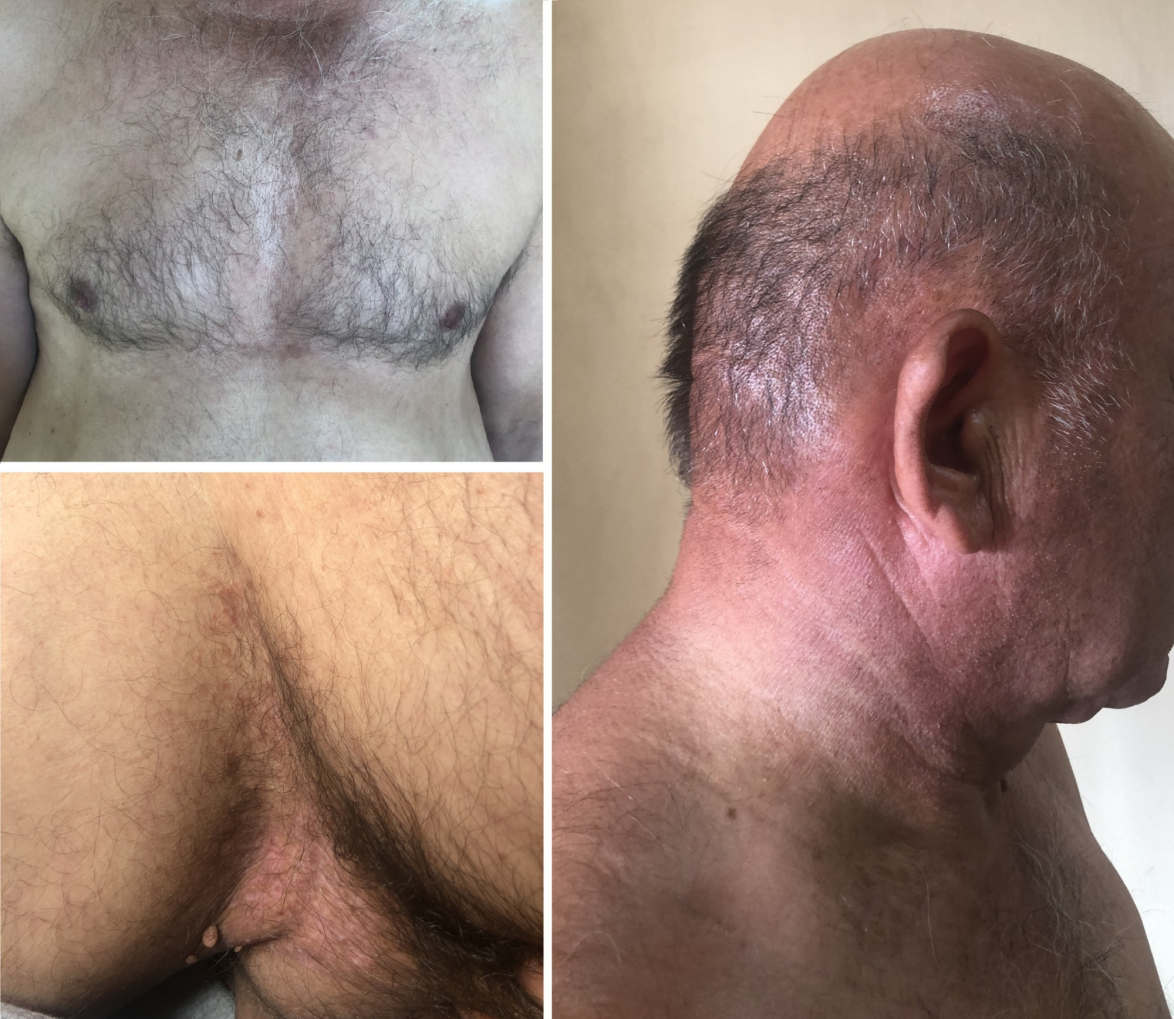
Outcome after two months of treatment. The hyperkeratotic papules and plaques resolved and the erosive flexural lesions are markedly improved.

Based on clinical and histopathological grounds, the diagnosis of DD with superinfected lesions was rendered. Intravenous antibiotic therapy with piperacillin plus tazobactam and ciprofloxacin was administered within the 10 days of hospitalization. Treatment with oral isotretinoin 20 mg per day was also initiated. The topical treatment consisted of antiseptic washes with chlorhexidine gluconate, fusidic acid 2% cream, ketoconazole 2% cream and emollients. After two months, at follow-up, the results were extremely satisfactory (Figure 3).

## Discussion

This case highlights the variable expressivity of Darier disease, with a clinical presentation too discrete to be identified. For nearly 40 years, the patient probably had a “forme fruste” (incomplete phenotypic expression) of DD and the classical features of the disease had a very late onset.

With the current knowledge, it is difficult to identify the mechanism behind the mild course of our patient and the late onset of the classical features of DD. This could represent a natural variability of symptoms in DD [1] or could suggest that the expression of this disease is modulated by many other environmental and other genetic factors [5]. For example, in a case of very subtle clinical presentation of DD in a skin type V individual (dark skin color according to Fitzpatrick skin phototype), the mutation analysis revealed a novel p.Leu590Pro mutation in the ATP2A2 gene [6].

Our differential diagnoses included severe seborrheic dermatitis, Hailey-Hailey disease and acrokeratosis verruciformis of Hopf. The presence of hand and nail involvement ruled out the diagnosis of severe seborrheic dermatitis. Due to the flexural involvement, Hailey-Hailey disease was considered, but, histologically, Hailey-Hailey is characterized by more massive acantholysis and less pronounced dyskeratosis [7]. The verrucous, flat-topped papules on the dorsa of the hands were clinically indistinguishable from acrokeratosis verruciformis, a condition allelic to Darier's disease [2]. Acrokeratosis verruciformis of Hopf (AVH) is caused by a pro602Leu mutation (P602L) in ATP2A2. It has clinical similarities with DD but it does not affect sebaceous areas, flexural sites, lacks greasy, malodorous papules, and does not involve oral mucosa [2]. Hands involvement with lesions similar to those of AVK is a frequent characteristic of patients with classical variant of Darier disease [8].

After excluding the above conditions, the diagnosis of DD was rendered on clinical and histopathological grounds. Owing to a lack of available laboratory equipment, we were not able to perform a mutation analysis of our patient.

Of note, an interesting theory states that on account of the high expressions of calcium pumps in the brain tissue, DD may be associated with neuropsychiatric disorders [9]. Recent reports have presented an association of dyskeratosis follicularis with psychiatric abnormalities, such as schizophrenia [10], intellectual impairment, deterioration of mental abilities, bipolar disorder [11] and depression [12]. Our patient underwent psychiatric evaluation and was not found to have any associated mental illnesses.

As for the treatment, there is no cure for Darier disease. The disease usually runs a chronic course with recurrent exacerbations, particularly triggered by heat, friction, UVB radiation or infections [3]. The goals of treatment are: trigger avoidance, control of skin appearance, alleviation of symptoms (e.g., itch, malodour), and prevention/treatment of superinfection. Oral retinoids have shown to be effective in producing significant clinical improvements, generally after a course of minimum three to four months [13,14]. Interestingly, in our patient, extremely satisfactory results were achieved after only two months of therapy.

Our patient continues to follow good personal care, specific hygiene norms, and oral retinoids therapy will be continued for long-term in order to prevent relapses [13,14]. Doses of oral isotretinoin will be adjusted at follow-up.

## Conclusions

This case highlights that a longstanding history of persistent, hyperkeratotic papules, from unknown origin, should prompt histopathological testing for Darier disease, even in the absence of classical findings. Due to its variable expressivity, dyskeratosis follicularis can present with clinical features too discrete to be identified. Delays in the diagnosis can result in severe exacerbations which prompt more aggressive treatment.
